# SHED-derived exosomes attenuate trigeminal neuralgia after CCI of the infraorbital nerve in mice via the miR-24-3p/IL-1R1/p-p38 MAPK pathway

**DOI:** 10.1186/s12951-023-02221-6

**Published:** 2023-11-29

**Authors:** Rong Guo, Yuxin Fang, Yuyao Zhang, Liu Liu, Na Li, Jintao Wu, Ming Yan, Zehan Li, Jinhua Yu

**Affiliations:** 1 Department of Endodontics, The Affiliated Stomatological Hospital of Nanjing Medical University, Shanghai Road, Nanjing, 210029 Jiangsu China; 2 Jiangsu Province Key Laboratory of Oral Diseases, Nanjing Medical University, 136 Hanzhong Road, Nanjing, 210029 Jiangsu China; 3 Jiangsu Province Engineering Research Center of Stomatological Translational Medicine, Nanjing Medical University, 136 Hanzhong Road, Nanjing, 210029 Jiangsu China

**Keywords:** Stem cells from human exfoliated deciduous teeth, Exosome, Trigeminal neuralgia, Microglia, miR-24-3p, IL1R1

## Abstract

**Background:**

Microglial activation in the spinal trigeminal nucleus (STN) plays a crucial role in the development of trigeminal neuralgia (TN). The involvement of adenosine monophosphate-activated protein kinase (AMPK) and N-methyl-D-aspartate receptor 1 (NMDAR1, NR1) in TN has been established. Initial evidence suggests that stem cells from human exfoliated deciduous teeth (SHED) have a potential therapeutic effect in attenuating TN. In this study, we propose that SHED-derived exosomes (SHED-Exos) may alleviate TN by inhibiting microglial activation. This study sought to assess the curative effect of SHED-Exos administrated through the tail vein on a unilateral infraorbital nerve chronic constriction injury (CCI-ION) model in mice to reveal the role of SHED-Exos in TN and further clarify the potential mechanism.

**Results:**

Animals subjected to CCI-ION were administered SHED-Exos extracted by differential ultracentrifugation. SHED-Exos significantly alleviated TN in CCI mice (increasing the mechanical threshold and reducing p-NR1) and suppressed microglial activation (indicated by the levels of TNF-α, IL-1β and IBA-1, as well as p-AMPK) in vivo and in vitro. Notably, SHED-Exos worked in a concentration dependent manner. Mechanistically, miR-24-3p-upregulated SHED-Exos exerted a more significant effect, while miR-24-3p-inhibited SHED-Exos had a weakened effect. Bioinformatics analysis and luciferase reporter assays were utilized for target gene prediction and verification between miR-24-3p and IL1R1. Moreover, miR-24-3p targeted the IL1R1/p-p38 MAPK pathway in microglia was increased in CCI mice, and participated in microglial activation in the STN.

**Conclusions:**

miR-24-3p-encapsulated SHED-Exos attenuated TN by suppressing microglial activation in the STN of CCI mice. Mechanistically, miR-24-3p blocked p-p38 MAPK signaling by targeting *IL1R1*. Theoretically, targeted delivery of miR-24-3p may offer a potential strategy for TN.

**Graphical Abstract:**

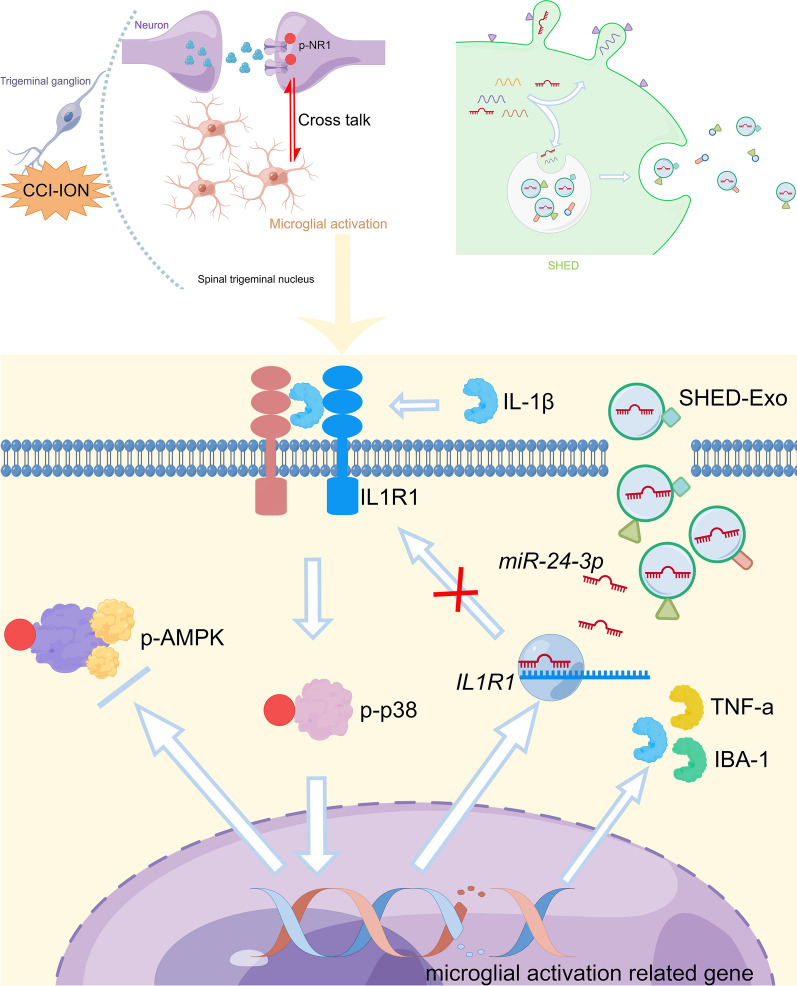

**Supplementary Information:**

The online version contains supplementary material available at 10.1186/s12951-023-02221-6.

## Background

Trigeminal neuralgia (TN) is a highly distressing condition that significantly affects the overall quality of life of patients [1]. TN can be diagnosed by the presence of localized pain in one or multiple branches of the trigeminal nerve, which is characterized by paroxysmal pain triggered by certain stimuli and is often described as a “shock” or an “electric sensation” [2]. The pathology and mechanism of TN are commonly attributed to neuropathic pain (NP), peripheral (trigeminal ganglion, TG) and central (spinal trigeminal nucleus, STN) sensitization, and neuroinflammation. However, further research is needed to uncover more precise mechanisms underlying this condition. Hyperalgesia is characterized by the activation of multiple neuronal receptors. One such receptor, the N-methyl-D-aspartate (NMDA) receptor, plays a significant role in pain development, with the NR1 subunit being susceptible to phosphorylation by PKC and PKA in the context of allodynia [3–5]. Adenosine monophosphate-activated protein kinase (AMPK) is a ubiquitous and highly conserved protein kinase endogenously activated by relatively reduced ATP levels (which signifies impaired energy status), and opposing roles in inflammation (an energy-intensive status) for AMPK have been proposed [6]. It has been identified as a vital regulator of neuronal function and plays key roles in the management of NP, including chronic constriction injury (CCI) to the unilateral infraorbital nerve (ION)-induced TN [4, 5], SNI-induced nerve hyperalgesia [7], CFA-induced inflammatory pain [8], and CCI-induced sciatic allodynia [9]. Therefore, the activation of AMPK could be considered a firmly established indicator of pain relief.

Growing evidence over the past decades suggests that microglial activation in the spinal trigeminal nucleus is responsible for generating NP during central sensitization [5, 10–12]. Traditional drugs targeting neuronal excitability (e.g., anticonvulsant agents) show limited efficacy and are not innocuous [13], which highlights a shift from regulating neuron plasticity to suppressing microglial activity for improved strategies. Microglial activation is implicated in the development of TN and enhances the responses of pain circuits and inflammatory cascades [14]. Accumulating evidence has emerged that proinflammatory cytokines (such as interleukin-1β (IL-1β) and tumor necrosis factor-α (TNF-α)) [15, 16], matrix metalloproteinase (MMP, such as MMP-9/2) [4], cell-surface receptors (such as CSF1R) [17] and intracellular transduction signaling (such as p38 MAPK, ERK, JNK) [5, 18] are involved in microglial activation. Targeting microglia is a potential strategy for pain hypersensitivity.

Stem cells from human exfoliated deciduous teeth (SHED), which are a type of mesenchymal stem cell originating from the embryonic neural crest, and their derivatives (including conditioned media (CM) and extracellular vesicles (EV)) have shown satisfactory neuroprotective and anti-inflammatory potential in neurological disorders [19–25]. In particular, studies have indicated that SHED possess the ability to alleviate TN [26, 27]. Compared to stem cell therapy, cell-free therapy has the superior profiles of low immunogenicity, incapability of self-replication [28], easier storage conditions, and biological activity and function that are not easily altered by the environment [29, 30]. EVs express multiple specific surface antigens (such as CD9, CD63, and TSG101) [31, 32] and show a saucer-shaped morphology under transmission electron microscopy (TEM). EV biological information (such as proteins, lipids, miRNAs, and mRNAs) from parent cells can be transmitted to recipient cells, thereby mediating cellular communication [33]. Exosomes originating as intraluminal vesicles are released from parent cells when a multivesicular body fuses with the plasma membrane and is 50–150 nm in diameter [32, 34]. The underlying mechanism by which microglial activation is regulated by SHED-Exos requires further investigation.

Interleukin-1 receptor type 1 (IL-1R1) is a subunit of a membrane receptor that has the ability to bind to the IL-1 family, thereby initiating an inflammatory response that typically involves the NF‑κB and MAPK pathways [35, 36]. In the acute-phase response, IL-1 receptor antagonist (IL-1Ra) can restrict the activation of IL-1 signaling by competitively binding to IL-1R1. However, in cases of chronic inflammation, the upregulation of IL-1Ra is insufficient, and the administration of exogenous IL-1Ra rarely reaches the central nervous system [37]. Theoretically, the suppression of IL1R1 levels may be a more effective and logical approach for controlling the inflammatory network in NP.

In this study, we conducted an investigation into the analgesic effect of exosomes derived from SHED in CCI-induced TN. Our focus was on suppressing microglial activation in the STN. Additionally, we explored the underlying mechanism by which SHED-Exos exerted their attenuating effect, specifically through the delivery of miR-24-3p-Exos. Ultimately, we demonstrated that the targeting of IL1R1-p38 MAPK signaling by miR-24-3p played a crucial role in microglial activation, leading to the alleviation of mechanical allodynia.

## Materials and methods

### Study design

**Fig. 1:**
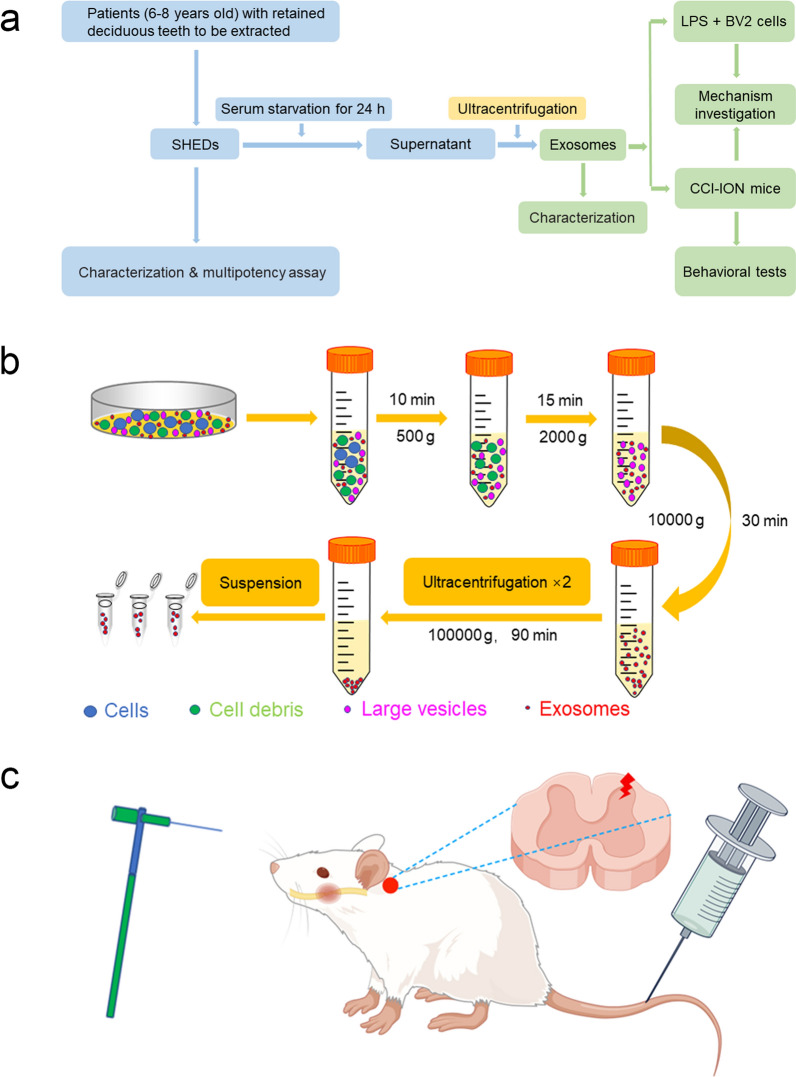
Conceptual schematic of this study. **a** Design flowchart. **b** Steps of differential ultracentrifugation. **c** Methodology of the animal experiment

The study design of our work is shown in Fig. 1a. Generally, healthy patients (6–8 years) with retained deciduous teeth were enrolled in Nanjing Medical University’s affiliated stomatology hospital, and their teeth were extracted for SHED isolation. Informed consent was obtained from all subjects and their parents. The supernatant of SHEDs cultured in serum-free medium was collected for the concentration of exosomes by differential ultracentrifugation. Protocols were consistent with previous research [38, 39], and a slight modification was made (Fig. 1b).

For the in vivo experiment, chronic constriction injury to the unilateral infraorbital nerve (CCI-ION) was conducted in mice to cause TN and sensitization in the STN. Behavioral tests with Von Frey filaments were performed to analyse the pain threshold. SHED-Exos were administered to mice intravenously (Fig. 1c).

For the in vitro experiment, lipopolysaccharide (LPS, 1 μg/mL) was added to complete medium to induce inflammatory BV-2 cells.

### Ethics statement

This study was approved by the Institutional Animal Care and Use Committee of Nanjing Medical University (Project No: IACUC-2104046) and by the Ethical Committee of Nanjing Medical University’s affiliated stomatology hospital (Project No.: (2018)202). All operation procedures were designed and strictly performed to minimize suffering.

### Animals

A total of 200 BALB/c male mice (20–22 g, 8 weeks old, at the start of the experiment, Experimental Animal Center at Nanjing Medical University) were housed in specific pathogen-free conditions with controlled temperature (22 ± 2 °C) and photoperiods (12 h light/dark cycle) and provided water and food ad libitum. All mice were allowed to acclimate to the environment for 3 days before inclusion in experiments and were randomly divided into groups (n = 7 per group).

### CCI-ION models

The CCI-ION model was established in mice as previously reported with slight modification [5]. All surgeries were performed aseptically under anaesthesia with 4% sodium pentobarbital (50 mg/kg, i.p., Sigma, USA). Skin preparation in the surgical area for a mouse in the prone position with a fixed head and limbs was conducted. A 5 mm skin incision was then made along the left infraorbital margin (1 cm from the lower eyelid) from proximal to distal, beginning at the end of the whisker pad. After blunt dissection of muscles, the infraorbital nerve was exposed, dissociated from the bone surface and loosely tied with 2 chromic gut ligatures (5–0, 2.5 mm apart), and then the incision was closed. Sham-operated mice received only skin incision and muscle dissection but no nerve contact or ligation.

### Von frey tests

The mechanical threshold of the whisker pad (the left infraorbital nerve receptive zone) was assessed with a series of calibrated Von Frey hairs (buckling weights were 0.008, 0.02, 0.04, 0.07, 0.16, 0.4, 0.6, 1, 1.4, 2 g, Aesthesio^®^, USA). Briefly, a series of Von Frey filaments ranging from 0.008 to 2 g were lightly applied the ipsilateral whisker pad, each filament was tested 5 times at 5 s intervals. The definition of a positive response is that the mouse has a brisk and active withdrawal of the head from the probing filament, and the definition of withdrawal threshold was the minimum stimulating force in grams corresponding to a filament that resulted in 3 or more positive responses in 5 successive applications [4, 5, 26, 40]. When testing, a mouse was shaved around the whisker pad and held upright in an experimenter’s hands for 5–10 min to make it calm, with its head exposed freely. Afterwards, the fibre filaments were lightly applied to the center of the whisker pad until a positive response was evoked. All behavioral testing was conducted with a double-blind method.

### SHED culture

Fresh extracted teeth were preimmersed in ice-cold phosphate buffered saline (PBS, Gibco, USA), and the pulp tissues were separated aseptically from hard tissue within 1 h. Briefly, the pulp was first digested in alpha minimum essential medium (α-MEM, Gibco, USA) containing 3 mg/mL type I collagenase (Gibco, USA) and 4 mg/mL trypsin (Gibco, USA) at 37 °C for 30 min, followed by digestion cessation and centrifugation (1000 rpm, 5 min). Afterwards, the cells were incubated in α-MEM containing 10% fetal bovine serum (FBS, HyClone, USA) and 1% streptomycin-penicillin (Gibco, USA) at 37 °C in a 5% CO_2_ incubator. The medium was replaced every 2 days until the confluence reached 95% for subculture at a ratio of 1:3. Third-passage cells were harvested for experiments.

### SHED phenotype and multipotency

The characteristics of SHED were evaluated, and the procedures were detailed in our previous study [41]. Briefly, positive (CD29, CD90. CD105) and negative (CD34, CD45) surface markers (BD Biosciences, USA) were utilized to verify the mesenchymal origin of SHED by flow cytometry (cells without antibody incubation as control). The multipotency of SHEDs was evaluated by inducing them to undergo osteogenic differentiation (shown as ALP & ARS staining), chondrogenic differentiation (shown as Alcian blue staining, adipogenic differentiation (shown as Oil Red O staining) and neurogenic differentiation (shown as immunofluorescence staining of βIII-tubulin and NeuN). Uninduced cells acted as a negative control.

### Concentration of SHED-Exos

Normalization strategies were pursued for the number of SHED-Exos. SHEDs were precultured with complete medium in a 150 mm dish and grown to 95% confluency, cleaned twice with PBS and incubated for 24 h in serum-free medium (Dulbecco’s modified Eagle’s high-glucose medium, high-glucose DMEM, ScienCell, China). A total of 35 mL of supernatant in each dish was harvested for differential ultracentrifugation (Fig. 1b). A Beckman Coulter Optima^™^ L-100XP ultracentrifuge with a Type SW 32 Ti 09U2498 rotor was used to pellet SHED-Exos. The resuspension of the resulting SHED-Exo pellets was conducted with 100 μL of PBS. The SHED-Exos suspension was immediately used for experiments or stored at -80℃.

### Identification of SHED-Exos

According to the updated MISEV of JSEV in 2018 and 2021 [32, 42], the morphological characteristics of SHED-Exos in this research were assessed through transmission electron microscopy (TEM) (JEM-1400 Flash, Japan). The diameter distribution and abundance of particles were evaluated by nanoparticle tracking analysis (NTA, ZetaView, Germany). Total protein amount measured by a BCA kit (Beyotime, China) was also used for quantification of SHED-Exos. Table 1 shows two methods for quantifying SHED-Exos. Specific proteins (CD9, TSG101 and CD63) and negative markers (CALNEXIN, which is located in the endoplasmic reticulum other than the plasma membrane or endosomes) of exosomes were analysed by western blot analysis in SHED-Exos and SHED-producing cells.

**Table 1:** Two methods for quantifying SHED-Exos

	BCA assay	NTA
Size of culture dish (mm)	150	150
Number of SHEDs	1.8 × 10^7^	1.8 × 10^7^
Volume of the medium (mL)	35	35
Volume of RIPA (μL)	100	–
Volume of PBS (μL)	–	100
Concentration	1.008 ± 0.127 (μg/μL)	2.3 × 10^11^ ± 8.66 × 10^9^ (particles/mL)

### SHED-Exos administration schedule

To evaluate amelioration by SHED-Exos in development of mechanical allodynia induced by CCI, SHED-Exos were administrated to mice through the tail vein immediately after surgery and on days 1, 2, and 3. To assess amelioration by SHED-Exos after reaching the hypersensitivity peak, mice administered SHED-Exos on days 14, 15, 16, 17, and 18. Mechanically, SHED-Exos were administrated intravenously on day 14, and tissues were collected 24 h later.

### BV-2 cell culture

Microglial BV-2 cells (ScienCell, China) were incubated at 37 °C in a humidified 5% CO_2_ incubator and cultured in DMEM containing 10% FBS and 1% penicillin‒streptomycin. The culture medium was replaced every 2 days, and subculture at a ratio of 1:3 was performed when the confluence reached 95%. Cells used for experiments were cultured in high-glucose DMEM complete medium (containing 0.5% FBS) and stimulated with LPS (1 μg/mL) with or without SHED-Exos.

### In vivo* distribution and *in vitro* internalization*

Protein quantification of the resulting exosomes by a BCA kit (Beyotime, China) was first implemented, after which 5 µL of PKH26 in 45 µL of Diluent C (UR52302, Umibio, China) was incubated with SHED-Exos in the dark for 10 min. Then, PBS was used to resuspend the Exos-PKH26 complex, and another round of ultracentrifugation was conducted.

For in vivo distribution assessment, CCI-ION mice were intravenously administered 100 μL of PKH26-labelled SHED-Exos on day 14 after CCI, and the left STN (C1-C2 spinal cervical dorsal horn) and TG were collected 24 h after exosome administration. The tissues were fixed in 4% paraformaldehyde (PFA) and dehydrated in 10, 20 and 30% sucrose solutions. Afterwards, tissues were embedded in OTC for frozen sectioning at 10 µm, and nuclei were stained with DAPI for fluorescent signal analysis (Leica, Germany).

For in vitro internalization, LPS-induced BV-2 cells were treated with Exos-PKH26 for 24 h and fixed for visualization by fluorescence microscopy (Leica, Germany).

### Quantitative reverse transcription and polymerase chain reaction (qRT‑PCR)

For tissue samples, the STN tissues ipsilaterally were immediately separated after the mice were deeply anaesthetized. Afterwards, STNs were subjected to ultrasonic fragmentation on ice for 2 min. Total RNA was extracted using TRIzol™ (Invitrogen, USA), and two separate RT Reagent Kits (Vazyme, China) for miRNA and total mRNA were used for reverse transcription. For cell samples, TRIzol reagent was added into dishes for cell lysis. qRT‒PCR was conducted in QuantStudio^™^ 7 Flex Real-Time PCR Systems (ABI, USA) and a Roche Light Cycler 480 sequence detection system (Roche Diagnostics, Switzerland). The procedure was set as reported [43]. The cycle threshold (CT) value measured the expression of genes (*miR-24-3p, U6, IL1R1, TNF-a, IL-1β, IBA-1 and GAPDH*). *GAPDH* and *U6* were used to normalize the mRNA expression and miR-24-3p, respectively. Relative gene expression was detected using the 2^−△△Ct^ relative expression method, and the primers used are listed in Table 2.

**Table 2:** Sense and antisense primers for qRT-PCR

Genes	Primers	Sequence (5′-3′)
*miR-24-3p*	Forward	GCGTGGCTCAGTTCAGCAG
	Reverse	AGTGCAGGGTCCGAGGTATT
*U6*	Forward	CTCGCTTCGGCAGCACA
	Reverse	AACGCTTCACGAATTTGCGT
*mIL1R1*	Forward	GTGCTACTGGGGCTCATTTGT
	Reverse	GGAGTAAGAGGACACTTGCGAAT
*mIL-1β*	Forward	GCAACTGTTCCTGAACTCAACT
	Reverse	ATCTTTTGGGGTCCGTCAACT
*mTNF-a*	Forward	CTGAACTTCGGGGTGATCGG
	Reverse	GGCTTGTCACTCGAATTTTGAGA
*mIBA-1*	Forward	ATCAACAAGCAATTCCTCGATGA
	Reverse	CAGCATTCGCTTCAAGGACATA
*mGAPDH*	Forward	AGGTCGGTGTGAACGGATTTG
	Reverse	TGTAGACCATGTAGTTGAGGTCA

### Western blot analysis

After the mice were deeply anaesthetized, the STN tissues ipsilaterally were immediately separated and homogenized in RIPA lysis buffer (Beyotime, China) containing 1 mM PMSF (Beyotime, China) and 2 mM phosphatase inhibitors (Beyotime, China). Whole-cell protein extraction of BV-2 cells was carried out for in vitro assessment. Protein concentrations were quantified using a BCA assay (Beyotime, China). Protein samples were loaded on and segregated by SDS‒PAGE and electrotransferred onto PVDF membranes (Millipore, USA). The membranes were blocked with 5% bovine serum albumin (BSA) and probed overnight at 4 °C with primary antibodies against p-AMPK (Thr183/172, 1:1000, ab133448, Abcam, USA), AMPK (1:1000, ab207442, Abcam, USA), p-NR1 (Ser896, 1:500, CY6380, Abways, China), IBA-1 (1:1000, #17198, Cell Signaling Technology, USA), IL-1β (1:500, #12242, Cell Signaling Technology, USA), TNF-α (1:500, #3707, Cell Signaling Technology, USA), IL1R1 (1:1000, sc-393998, Santa, USA), p-p38 (Thr180/182, 1:1000, #9216, Cell Signaling Technology, USA), and GAPDH (1:2000, 10494–1-AP, Proteintech, USA). Afterwards, the membranes were incubated with horseradish peroxidase (HRP)-conjugated secondary antibodies for 1 h, and enhanced chemiluminescence (ECL, Tiannneng, China) reagents were utilized for signal detection. Data were analysed with ImageJ software.

### Immunofluorescence

For fluorescent immunohistochemistry, after deep anaesthesia, the C1-C2 cervical spinal cord segment where the STN is located and the left TG were immediately isolated and fixed in 4% PFA overnight. After orderly dehydration in 10, 20 and 30% sucrose solution, the ipsilateral STN tissues were embedded in OTC for frozen sectioning at 10 µm. In general, sections were permeabilized with Triton-100 (Beyotime, China) for 10 min and blocked with goat serum at 37 °C for 1 h. Diluted primary antibodies against IBA-1 (1:50), IL1R1 (1:50), and p-p38 (1:200) were used to probe tissue sections overnight at 4 °C, followed by washing and incubation with fluorescent secondary antibody at room temperature in the dark for 1 h. Finally, sections were sealed with antifade mounting medium containing DAPI (Beyotime, China) for fluorescence observation and capture (Leica, Germany).

For the immunocytochemistry assay, BV-2 cells were seeded onto coverslips in 12-well plates for experiments. Cells on coverslips were fixed and washed for subsequent blocking, incubation with antibodies, and fluorescence analysis.

### Transfection assay and expression control of miR-24-3p in SHED-Exos

SHEDs were transfected with miR-24-3p mimics/NC (50 nM, RiboBio, China) and inhibitor/INC (100 nM, RiboBio, China) using riboFECT™ CP transfection reagents (RiboBio, China) for 72 h, followed by culture in fresh complete medium until 90% confluence was reached. Cells were washed twice with PBS and incubated for 24 h in serum-free high-glucose DMEM. Finally, the supernatant was collected, and differential plus ultracentrifugation was performed as above to enrich miR-24-3p-Exos or miR-24-3p-inhib-Exos (NC-Exos and INC-Exos as the control). qRT‒PCR was conducted to detect transfection efficiency, and the phenotypes of exosomes in the groups were evaluated.

### Bioinformatic analysis of gene targeting

Target gene intersections of miR-24-3p were retrieved online in the miRDB (https://mirdb.org/faq.html), PicTar (https://pictar.mdc-berlin.de), TargetScan (https://www.targetscan.org/vert_80) and miRWalk (http://mirwalk.umm.uni-heidelberg.de) databases and output as a Venn diagram through the R language. Gene Ontology (GO) and Kyoto Encyclopedia of Genes and Genomes (KEGG) enrichment analyses were carried out by the DAVID tool (https://david.ncifcrf.gov) online to predict the functions and signaling pathways of miR-24-3p targets. Binding sequences of *miR-24-3p* and *IL1R1* mRNA 3’UTR were verified in the TargetScan database.

### Luciferase reporter assay

A luciferase reporter assay was performed to define the binding of *IL1R1* and *miR-24-3p*. In brief, the wild-type and mutant binding sequences (IL1R1-WT and IL1R1-MUT) were constructed via GV272 vectors inserted into firefly luciferase genes (Genechem, China). miR-24-3p mimics/NC intervened HEK-293 T cells (Sciencell, China) were seeded into a 24-well plate and *IL1R1* plasmids (100 ng/well)/renilla luciferase (20 ng/well) plasmid were transfected into HEK-293 T cells using Lipofectamine 2000 (Invitrogen, USA) for 48 h. The luciferase activity was detected by Dual-Luciferase Reporter Assay System (Promega, USA) and the data were shown as the ratio of firefly to renilla luciferase activity.

### IL1R1 expression silencing and overexpression

The upregulated expression of IL1R1 in LPS-induced BV-2 cells was first determined. IL1R1 was stably silenced in BV-2 cells by small interfering RNA (si-IL1R1, 50 nM, Ribobio, China) for 24 h, followed by stimulation with LPS (1 μg/mL) for 2 h, and the biological function was verified in vitro. In the rescue experiment, BV-2 cells were transfected with constructed plasmids of IL1R1 and negative control (IL1R1-Over/NC-Over) (Genechem, China) using Lipofectamine 2000 reagent. The 5'Chol + 2'OMe-modified siRNA of IL1R1 was hydrated with PBS (10 nmol/100 μL) and intravenously administered to CCI mice for in vivo verification.

### Cell proliferation assay

A cell counting kit-8 (CCK-8, A311-01, Vazyme, China) assay was conducted to assess the effect of SHED-Exos on the proliferation ability of BV-2 cells. BV-2 cells were seeded in a 96-well plate (5000 cells/well) until the cells were attached, followed by incubation with LPS (1 μg/mL) and LPS + SHED-Exos (BV-2 cells without intervention as an internal control). A mixture of 10 μL CCK-8 reagent and 90 μL DMEM was added to each well at 0, 4, 8, 12, 24 and 48 h, and the OD values (absorbance at 450 nm) were recorded after 2 h of incubation.

### Cell cycle assay

To evaluate the role of miR-24-3p and IL-1R1 in the cell cycle of BV-2 cells, PI flow cytometry was conducted. BV-2 cells (10^6^ per dish) transfected with miR-24-3p mimics/NC or si-IL1R1/si-NC were seeded into dishes (60 mm) and stimulated by LPS until reaching 90% confluence, followed by probing with PI (550825, BD, USA) for 15 min and then evaluation with a FACScan flow cytometer (BD Biosciences, USA). *FlowJo* software was used for data analysis.

### Statistical analysis

The quantitative data are shown as the mean ± standard deviation (SD). Differences in quantitative statistics between the groups were determined by Student’s t test or one-way analysis of variance (ANOVA). The differences of behavioral responses in latency over time among groups were tested with two-way ANOVA. Bonferroni post hoc corrections were conducted for all ANOVA models. Differences in constituent ratios in this study were compared by the chi-square test. All statistical analyses were performed by *GraphPad Prism 5.0*. A *p* value of < 0.05 was considered statistically significant.

## Results

### Characterization of SHEDs and SHED-Exos

A retained deciduous tooth was diagnosed by the panoramic radiograph (Additional file 1: Fig. S1a (i)) and then extracted for primary cell culture. The primary and passage 3 cells are shown in Additional file 1: Fig. S1a (ii) and (iii). The cells were positive for the mesenchymal stem cell biomarkers CD29, CD90, and CD105 but negative for the hematopoietic stem cell biomarkers CD34 and CD45 (Additional file 1: Fig. S1b), which proved their mesenchymal origin. For multipotency, the cells could be induced into osteoblasts, chondrocytes, adipocytes and neurocytes, as shown by ALP staining/mineralization formation (Additional file 1: Fig. S1c (i), (ii)), acidic mucopolysaccharide staining by Alcian blue staining (Additional file 1: Fig. S1c (iii)), lipid droplets dyed with Oil red O (Additional file 1: Fig. S1c (iv)) and enhanced fluorescence intensity of the neuronal markers NeuN and βIII-tubulin (Additional file 1: Fig. S1c (v)).

**Fig. 2:**
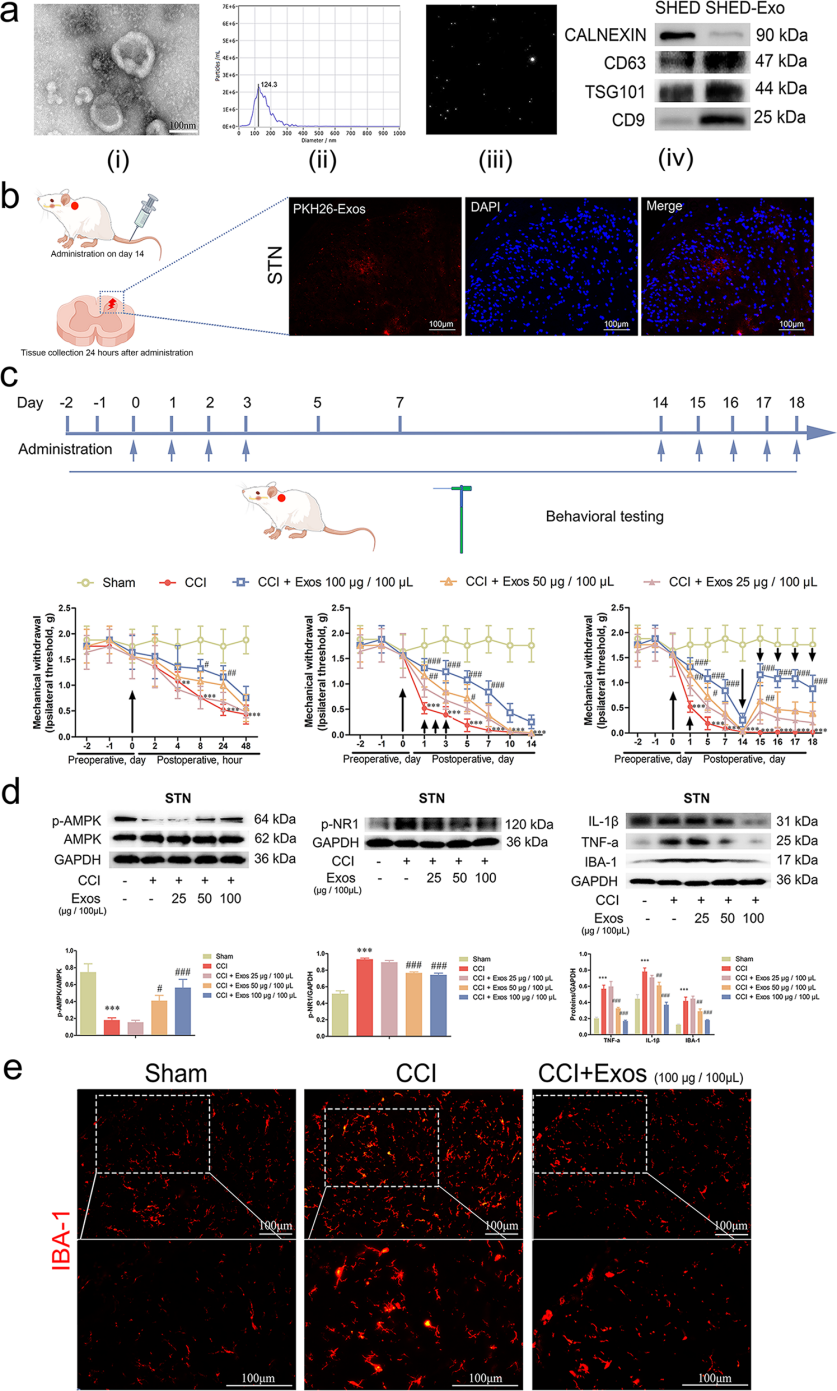
SHED-Exos alleviate mechanical allodynia in CCI mice by suppressing microglial activation. **a** Characteristics of SHED-Exos. **(i)** Morphology of SHED-Exos through TEM (scale bar: 100 nm), **(ii)** particle size distribution of SHED-Exos detected by NTA, **(iii)** tracked particles in NTA, **(iv)** the SHED-Exo markers CD9, CD63 and TSG101 and the SHED marker CALNEXIN were detected by western blot. **b** PKH26-labelled SHED-Exos were distributed in the ipsilateral STN (scale bar: 100 μm). **c (i)** Single-dose administration of SHED-Exos (25, 50, and 100 μg/100 μL, i.v.) attenuated CCI-induced mechanical allodynia within 24 h after surgery, **(ii)** consecutive administration of SHED-Exos (25, 50, and 100 μg/100 μL, i.v.) on days 0, 1, 2, and 3 after surgery ameliorated mechanical allodynia for 1 week, and **(iii)** readministration of SHED-Exos (25, 50, and 100 μg/100 μL, i.v.) on days 14–18 attenuated mechanical allodynia for 5 days. (The administration time is indicated by the arrows). **d** Protein levels of p-AMPK, p-NR1, IL-1β, TNF-a and IBA-1 in the STN were examined by western blot analysis and gray value analysis. **e** Immunofluorescence experiments revealed IBA-1 microglia in the ipsilateral STN of CCI mice (the C1–2 spinal dorsal horn) (scale bar: 100 μm). (**p < 0.01, ***p < 0.001 vs. sham group; #*p* < 0.05, ##*p* < 0.01, ###*p* < 0.001 vs. CCI group)

Particles concentrated by differential ultracentrifugation presented a characteristic saucer-like shape, as shown by TEM (Fig. 2a (i)). The particle diameter distribution was mainly 50–150 nm with a peak particle size of 124.3 nm in NTA detection (Fig. 2a (ii)). The particle tracking image is shown in Fig. 2a (iii). Compared with protein lysates from parent cells, the total protein of exosomes contained massive amounts of CD63, TSG101 and CD9, but not CALNEXIN (Fig. 2a (iv)). The aforementioned results suggested that the SHED-derived particles extracted in our study were exosomes. Additionally, protein quantification showed that the concentration of SHED-Exos was approximately 1.008 ± 0.127 μg/μL, and particle quantification showed that the concentration of SHED-Exos was approximately 2.3 × 10^11^ ± 8.66 × 10^9^ particles/mL (Table 1). A BCA assay for protein quantification of SHED-Exos was used in subsequent experiments.

### SHED-Exos alleviated mechanical allodynia in CCI mice by suppressing glial activation

All mice survived after surgery in the present study. After administration, PKH26-labelled SHED-Exos were distributed in the left STN (Fig. 2b) and TG (Additional file 2: Fig. S2a). The mice that underwent CCI-ION exhibited mechanical allodynia of the whisker pad starting from 4 h after the surgery, as evidenced by behavioral tests. This mechanical withdrawal threshold was significantly reduced and maintained at a low level until day 14 postsurgery (*p* < 0.001, Fig. 2c). Remarkably, administration of SHED-Exos (25, 50, and 100 μg/100 μL, i.v.) contributed to the recovery of pain-related behaviors in a concentration-dependent manner, and SHED-Exos at 100 μg exerted prominent and stable alleviation (Fig. 2c). Specifically, immediate postoperative administration of SHED-Exos at 100 μg significantly increased mechanical withdrawal at 8 and 24 h postoperative (*p* < 0.05), and continuous administration at 24 h, 48 h and 72 h contributed to lasting effects for 1 week (*p* < 0.001). Furthermore, mechanical allodynia was effectively reversed through the continuous administration of SHED-Exos (100 μg) on postoperative days 14, 15, 16, 17, and 18. To provide evidence of the remodelling of neuronal excitability and synaptic efficacy by SHED-Exos, the level of phosphorylated NR1 in the STN was measured. There was a significant increase in p-NR1 in CCI models (*p* < 0.001), but this upregulation was rescued by the administration of SHED-Exos (50 and 100 μg) (*p* < 0.001).

To investigate the impact of SHED-Exos on AMPK activity, the phosphorylated levels of AMPK in the STN were measured, as previous studies have consistently demonstrated a negative correlation between AMPK activation and neuroinflammation. Our results indicated that p-AMPK was inhibited in CCI mice (*p* < 0.001), while SHED-Exos reactivated AMPK in a dose-dependent manner (*p* < 0.05). Microglial activation in the STN promotes central sensitization, and proinflammatory cytokines play classical roles in NP by stimulating microglia or neurons. Western blot assays demonstrated that the increased expression of IBA-1, TNF-a and IL-1β in the STN of CCI mice suggested microglial activation (*p* < 0.001), and SHED-Exos suppressed this activation, as shown by the decrease in IBA-1, TNF-a and IL-1β (*p* < 0.01). (Fig. 2d). Furthermore, SHED-Exos (100 μg) decreased the fluorescence intensity of IBA-1 in the STN compared to that in the CCI group (Fig. 2e).

In summary, the concentration-dependent inhibition of mechanical allodynia and microglial activation by SHED-Exos in CCI mice, as well as the dose-dependent activation of AMPK by SHED-Exos, were observed.

### SHED-Exos suppressed activation of BV-2 cells

**Fig. 3:**
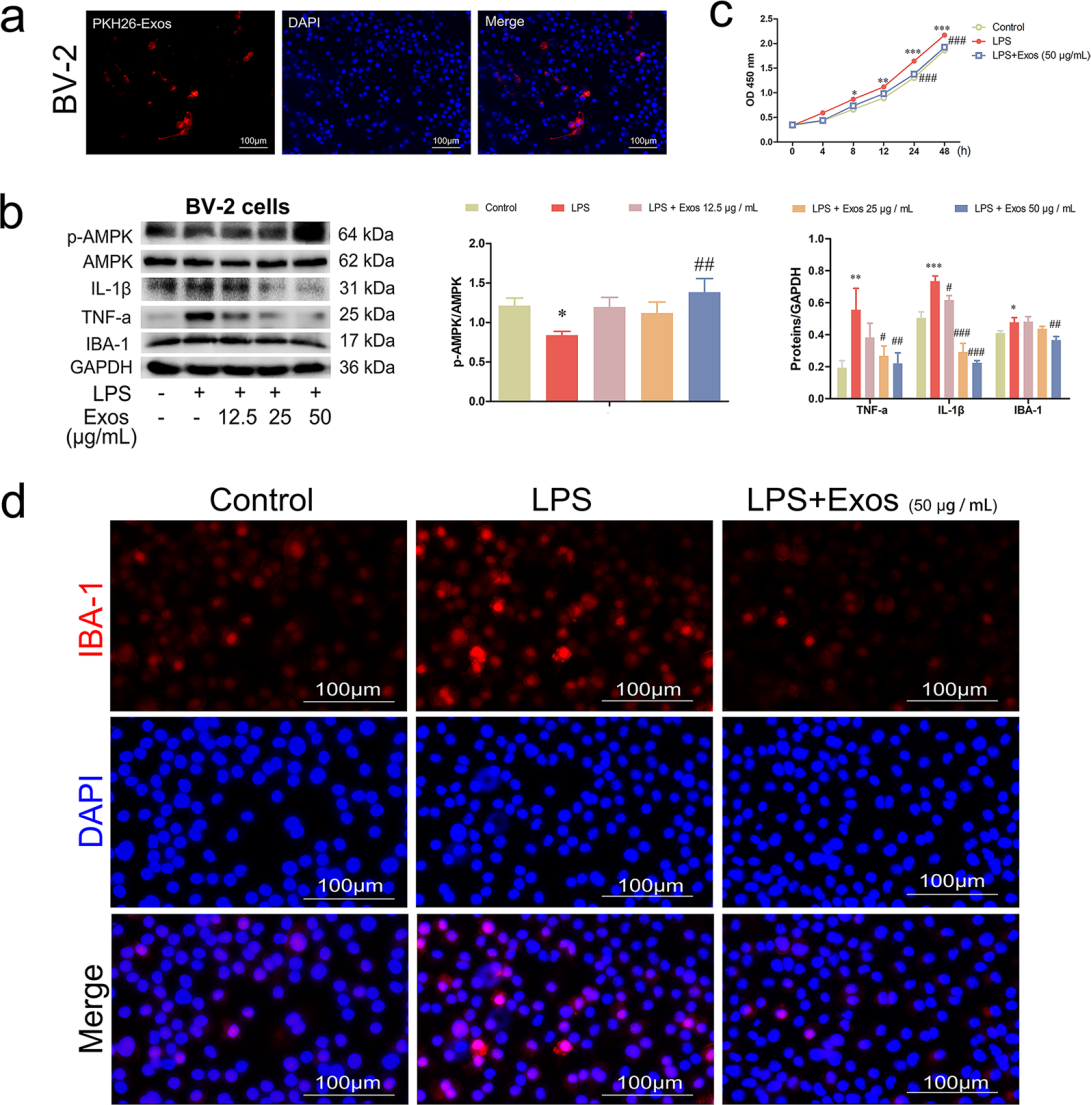
SHED-Exos suppressed the activation of BV-2 cells.** a** PKH26-labelled SHED-Exos were internalized by BV-2 cells (scale bar: 100 μm). **b** Expression levels of p-AMPK, IL-1β, TNF-a and IBA-1 in BV-2 cells were examined by western blot analysis and gray value analysis. **c** Results of the CCK-8 assay. **d** Immunofluorescence staining of IBA-1 in BV-2 cells. (*p < 0.05, **p < 0.01, ***p < 0.001 vs. Control group; #*p* < 0.05, ##*p* < 0.01, ###*p* < 0.001 vs. LPS group)

We further sought to expose cultured BV-2 cells to LPS (1 μg/mL) to replicate the impact of inflammation in an in vitro setting. First, we observed that PKH26-labelled SHED-Exos were internalized by LPS-induced BV-2 cells (Fig. 3a). As shown by the levels of p-AMPK, IL-1β, TNF-a and IBA-1, the expression of p-AMPK was upregulated and BV-2 cell activation was downregulated in a dose-dependent manner (SHED-Exos, 12.5, 25, 50 μg/mL), which was consistent with the in vivo results (*p* < 0.05, Fig. 3b). Moreover, the results of the CCK-8 assay showed inhibited proliferation in SHED-Exo-treated inflammatory BV-2 cells compared with the enhanced proliferation in stimulated BV-2 cells (*p* < 0.001, Fig. 3c). The immunofluorescent results showed that SHED-Exos (50 μg/mL) inhibited the expression of IBA-1 (Fig. 3d).

### Exosomes encapsulating miR-24-3p alleviated mechanical allodynia and suppressed microglial activation

**Fig. 4:**
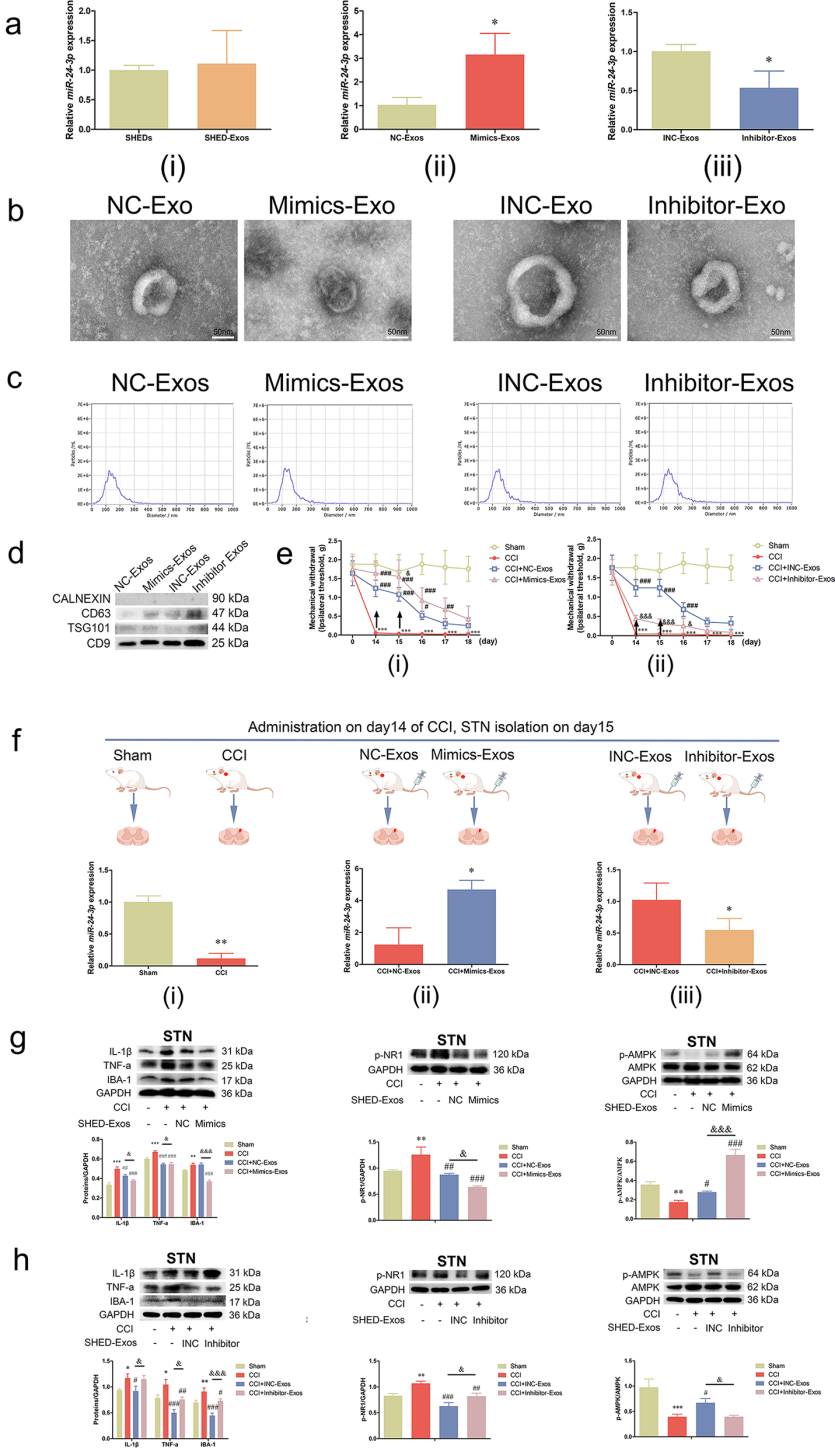
Exosome-encapsulated miR-24-3p alleviated mechanical allodynia and suppressed microglial activation. **a** **(i)** Expression of *miR-24-3p* by qRT‒PCR in SHEDs and SHED-Exos, **(ii)** expression of *miR-24-3p* was increased in SHED-Exos when miR-24-3p was overexpressed in SHEDs, and **(iii)** expression of *miR-24-3p* was decreased in SHED-Exos when miR-24-3p was inhibited in SHEDs. **b** TEM images showing the morphological characteristics of NC/Mimic-Exos and INC/Inhibitor-Exos (scale bar: 50 nm). **c** The size distributions of NC/Mimic-Exos and INC/Inhibitor-Exos by NTA. **d** Western blotting of CD9, CD63, TSG101 and CALNEXIN on NC/Mimic-Exos and INC/Inhibitor-Exos was conducted. **e (i)** Administration of mimics-Exos (100 μg/100 μL, i.v.) on days 14 and 15 after surgery significantly attenuated mechanical allodynia on day 15 compared with NC-Exos (100 μg/100 μL, i.v.), **(ii)** whereas administration of inhibitor-Exos (100 μg/100 μL, i.v.) reversed the analgesic effect on days 14, 15, and 16. **f (i)** Expression of *miR-24-3p* in sham/CCI mice, **(ii)** expression of *miR-24-3p* in CCI mice after administration of NC/mimic-Exos, and **(iii)** expression of *miR-24-3p* in CCI mice after administration of INC/inhibitor-Exos. **g** Protein levels of IL-1β, TNF-a, IBA-1, p-NR1 and p-AMPK in the STN of the sham/CCI/CCI + NC-Exos/CCI + Mimics-Exos groups were examined by western blot analysis and gray value analysis. **h** Protein levels of IL-1β, TNF-a, IBA-1, p-NR1 and p-AMPK in the STN of the sham/CCI/CCI + INC-Exos/CCI + Inhibitor-Exos groups were examined by western blot analysis and gray value analysis. (*p < 0.05, **p < 0.01, ***p < 0.001 vs. Sham group; #*p* < 0.05, ##*p* < 0.01, ###*p* < 0.001 vs. CCI group; &*p* < 0.05, &&*p* < 0.01, &&&*p* < 0.001 vs. NC/INC-Exos groups)

The abundantly expressed miRNAs in SHED-Exos via miRNA microarray analysis have been reported [39]. To further clarify the mechanism of action of SHED-Exos in alleviating pain, we focused on miR-24-3p (one of the top 10 miRNAs) that could be potentially associated with neuroinflammation by retrieving previous studies. As shown in Fig. 4a (i), miR-24-3p was expressed in both SHEDs and SHED-Exos. The expression levels of miR-24-3p in SHED-Exos were found to be significantly altered, either upregulated or downregulated, following transfection with mimics or inhibitor (Fig. 4a (ii)(iii)). Additionally, the phenotypes of exosomes secreted from SHEDs transfected with NC/mimics/INC/inhibitor were evaluated, including TEM morphology (Fig. 4b), size distribution by NTA (Fig. 4c) and high expression levels of CD63/TSG101/CD9 by western blot analysis (Fig. 4d). Behavioral tests were conducted to assess the analgesic effect of miR-24-3p after administration of mimics-Exos or inhibitor-Exos (100 μg/100 μL, i.v.) on days 14 and 15. Our results suggested that Exos with abundant miR-24-3p markedly improved the withdrawal threshold compared with the NC group (*p* < 0.05, Fig. 4e(i)), whereas miR-24-3p-inhib-Exos reversed the effects of SHED-Exos (*p* < 0.05, Fig. 4e(ii)). Interestingly, miR-24-3p was detected in the STN, and the levels of miR-24-3p were decreased in the STN of CCI mice compared to that of sham mice (*p* < 0.01, Fig. 4f(i)). Administration of mimics-Exos induced an increase in miR-24-3p, but inhibitor-Exos reduced the expression of miR-24-3p (*p* < 0.05, Fig. 4f(ii), (iii)). More importantly, miR-24-3p-mimics-Exos significantly inhibited microglial activation (reduced IBA-1, IL-1β and TNF-a) and levels of p-NR1 while enhancing the phosphorylation of AMPK (*p* < 0.05, Fig. 4g). In the rescue experiment, the results were reversed when miR-24-3p was inhibited in SHED-Exos (*p* < 0.05, Fig. 4h). Furthermore, miR-24-3p induced alterations in the cell cycle of the inflammatory BV-2 cells, resulting in a reduction of the S + G2/M phase in the mimics groups and an elevation of the S + G2/M phase in the inhibitor groups (*p* < 0.0001, Additional file 2: Fig. S2b, c, Additional file 3: Table S1, Additional file 4: Table S2). Notably, SHED-Exos-derived miR-24-3p exerted pivotal roles in alleviating TN in CCI mice.

### miR-24-3p targeted IL1R1 on the basis of bioinformatic analysis

**Fig. 5:**
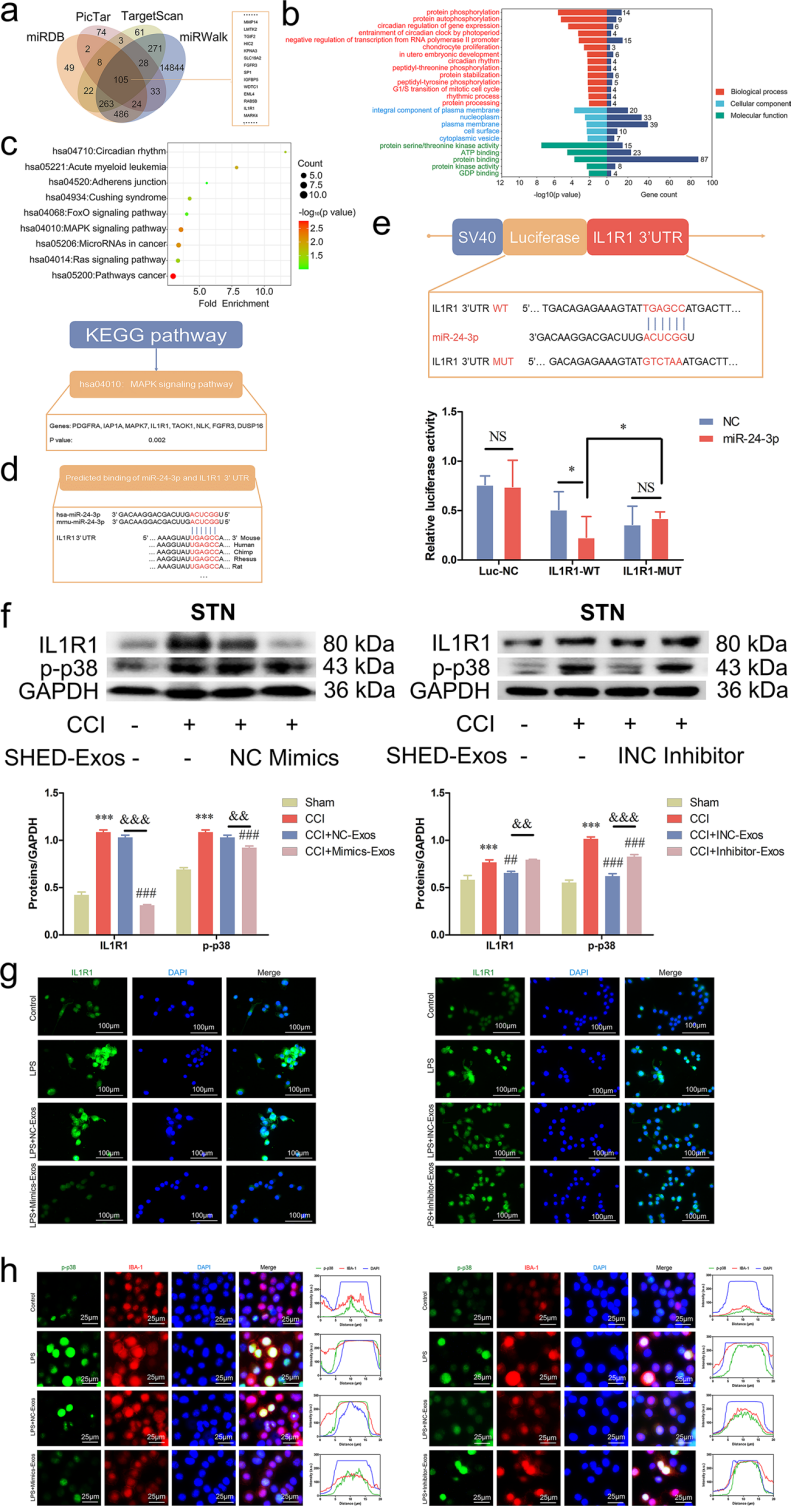
miR-24-3p targeted IL1R1 on the basis of bioinformatic analysis. **a** Venn diagram of target gene prediction databases (miRDB, PicTar, TargetScan and miRWalk) for gene targeting of miR-24-3p. **b** Terms of GO enrichment analysis sorted by *p* value (*p* < 0.01). **c** KEGG enrichment analysis of target genes involved in significant signaling pathways and the list of targets in the MAPK signaling pathway. **d** The sequences of *miR-24-3p* and the binding sequences between *miR-24-3p* and the *IL1R1* 3’UTR. **e** Plasmid construction of *IL1R1* 3’UTR wild/mutation type and dual luciferase reporter assay in 293 T cells further illustrated binding between *miR-24-3p* and *IL1R1*. **f** Administration of mimics-Exos (100 μg/100 μL, i.v.) on day 14 after surgery significantly suppressed the expression of IL1R1 and p-p38 in the STN of CCI mice compared with NC-Exos (100 μg/100 μL, i.v.), whereas administration of inhibitor-Exos (100 μg/100 μL, i.v.) reversed this effect. **g** Fluorescent immunocytochemistry assay showed IL1R1 BV-2 cells of Control/LPS/LPS + NC-Exos/LPS + Mimics-Exos groups and Control/LPS/LPS + INC-Exos/LPS + Inhibitor-Exos groups (scale bar: 100 μm). **h** Colocalization of p-p38 and IBA-1 in BV-2 cells from the Control/LPS/LPS + NC-Exos/LPS + Mimics-Exos and Control/LPS/LPS + INC-Exos/LPS + Inhibitor-Exos groups (scale bar: 25 μm). (***p < 0.001 vs. Sham group; ##*p* < 0.01, ###*p* < 0.001 vs. CCI group; &&*p* < 0.01, &&&*p* < 0.001 vs. NC/INC-Exos groups)

A total of 105 collective target genes of miR-24-3p were gathered in the miRDB, PicTar, miRWalk and TargetScan databases (Fig. 5a), and these genes were mainly involved in the molecular function of protein binding, cellular component of plasma membrane and biological process of cell proliferation according to GO annotation analysis (Fig. 5b). Moreover, the targets specifically directed towards the MAPK pathway were screened, as indicated by the KEGG pathway analysis depicted in Fig. 5c. *IL1R1* is one of the key upstream molecules in the MAPK signaling pathway, and we verified that the mRNA level of *IL1R1* in BV-2 cells was significantly inhibited by SHED-Exos (Additional file 2: Fig. S2d). According to the TargetScan database, the sequences of *miR-24-3p* and *IL1R1* are conserved between species (Fig. 5d). To test the direct binding of miR-24-3p and *IL1R1*, plasmids containing IL1R1-WT and IL1R1-MUT were constructed and transfected into 293 T cells, and the dual luciferase reporter gene assay showed that miR-24-3p significantly inhibited the luciferase activity of the IL1R1-WT reporter gene (Fig. 5e). Furthermore, the expression of IL1R1 and p-p38 MAPK was suppressed in CCI mice administered miR-24-3p-overexpressing SHED-Exos but enhanced in those administered miR-24-3p-inhibited SHED-Exos, which illustrated that miR-24-3p targeted the IL1R1-p38 MAPK axis (*p* < 0.01, Fig. 5f). In LPS-stimulated BV-2 cells, immunofluorescent results further proved that IL1R1 and p-p38 MAPK were regulated by miR-24-3p (Fig. 5g, h).

### Silencing IL1R1 alleviated mechanical allodynia and suppressed microglial activation

**Fig. 6:**
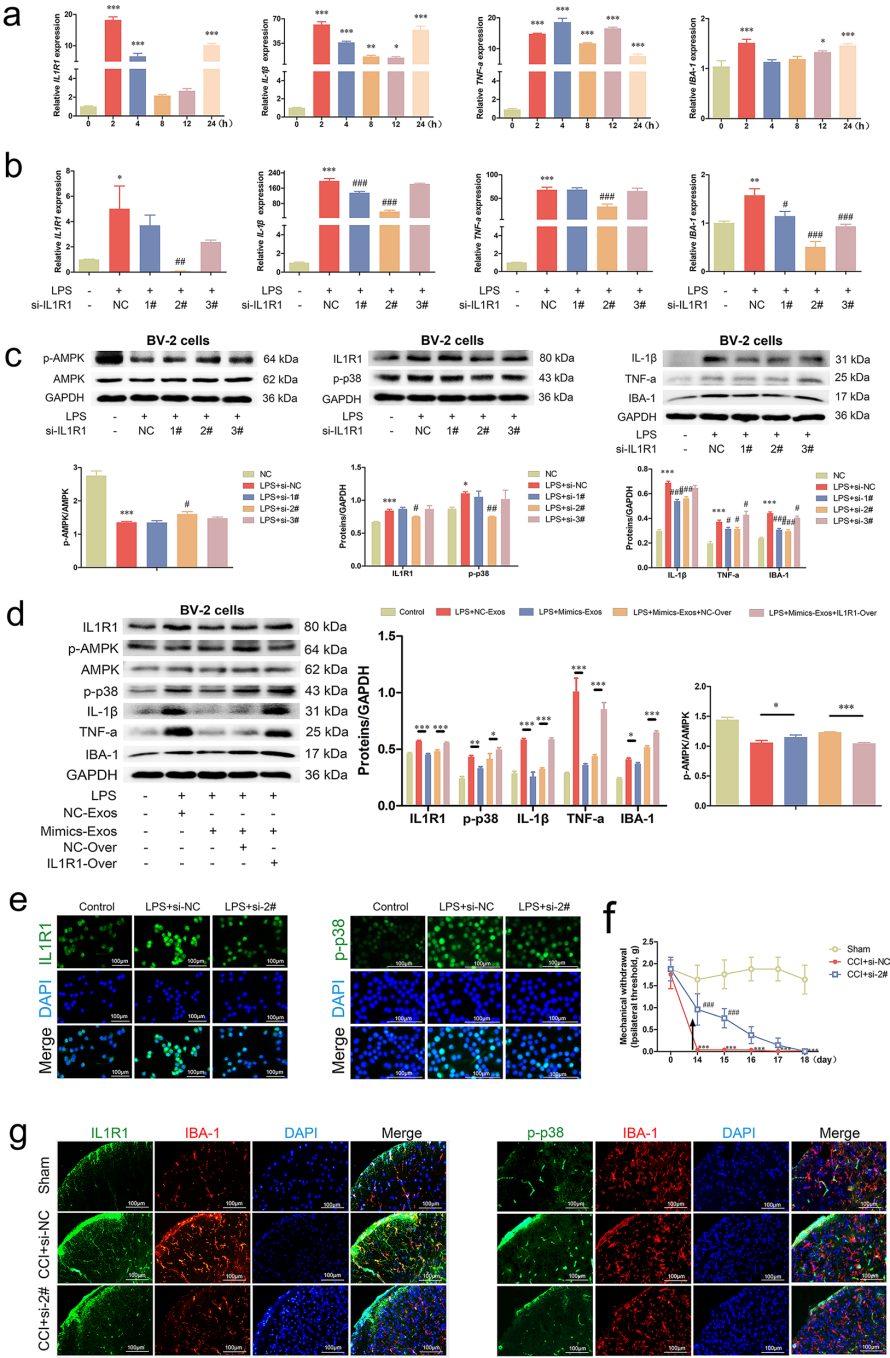
*Silencing IL1R1* alleviated mechanical allodynia and suppressed microglial activation. **a** Expression of *IL1R1*, *IL-1β*, *TNF-a* and *IBA-1* by qRT‒PCR in BV-2 cells after stimulation for 0, 2, 4, 8, 12, and 24 h. **b** qRT‒PCR examination showed that silencing IL-1R1 suppressed the mRNA expression of *IL1R1*, *IL-1β*, *TNF-a* and *IBA-1* in stimulated BV-2 cells. **c** Western blot analysis revealed that blocking the IL-1R1/p-p38 MAPK pathway activated AMPK and suppressed the protein levels of IL1R1, IL-1β, TNF-a and IBA-1 in impaired BV-2 cells. **d** Protein levels of IL1R1, p-AMPK, p-p38, IL-1β, TNF-a and IBA-1 in the rescue experiment. **e** Immunofluorescence experiments indicated that silencing IL-1R1 contributed to a decrease in IL-1R1 and p-p38 in LPS-stimulated BV-2 cells. **f** Mechanical allodynia was significantly attenuated on days 14 and 15 in CCI mice treated with si-IL1R1 #2 (10 nmol, i.v.) (scale bar: 100 μm). **g** Fluorescent colocalization analysis of IL1R1/IBA-1 and p-p38/IBA-1 in the STN revealed that administration of si-IL1R1 #2 (10 nmol, i.v.) inhibited the expression of IL1R1, p-p38 and IBA-1 (scale bar: 100 μm). (*p < 0.05, **p < 0.01, ***p < 0.001 vs. Control/Sham groups; #*p* < 0.05, ##*p* < 0.01, ###*p* < 0.001 vs. LPS/CCI groups)

To examine the involvement of IL1R1 in microglia, an experiment was conducted to silence IL1R1 both in vitro and in vivo. First, BV-2 cells were exposed to LPS (1 μg/mL) for 24 h, and the mRNA levels of IL1R1 were measured. The findings revealed that the expression of *IL1R1* exhibited consistent fluctuations, with a notable increase at 2 and 4 h (*p* < 0.001), a return to baseline levels at 8 and 12 h, and a significant upregulation at 24 h (*p* < 0.001). In addition, *IBA-1* showed a similar expression pattern. However, the mRNA levels of IL-1β and TNF-a were maintained at high levels from 2 to 24 h (*p* < 0.05). (Fig. 6a). Intriguingly, miR-24-3p was expressed in BV-2 cells but significantly decreased at 24 h (*p* < 0.001, Additional file 2: Fig. S2e). Thus, stimulation for 2 h after transfection with si-IL1R1 in BV-2 cells was performed. Our results demonstrated that si-IL1R1 (#2) prominently reduced the mRNA and protein upregulation of IL1R1, IL-1β, TNF-a and IBA-1 in inflammatory BV-2 cells (*p* < 0.05, Fig. 6b, c). The involvement of miR-24-3p and IL1R1 in the activation of BV-2 cells was reconfirmed by rescue assays (p < 0.05, Fig. 6d). In addition, the fluorescence intensity of IL1R1 and p-p38 was decreased (Fig. 6e). Conversely, activation of AMPK and levels of miR-24-3p were promoted in IL1R1-silenced BV-2 cells (*p* < 0.05, Fig. 6c; *p* < 0.05, Additional file 2: Fig. S2f). Additionally, the cell cycle of BV-2 cells transfected with si-IL1R1 (#2) was changed under inflammatory conditions, with a decrease in the S + G2/M phase in the si-IL1R1 groups (*p* < 0.05, Additional file 2: Fig. S2h, Additional file 5: Table S3).

To validate the in vivo effect of IL1R1, 5'Chol + 2'OMe-modified si-IL1R1 (2#, 10 nmol/100 μL, i.v.) was administered to mice 14 days after CCI. The transfection efficiency of si-IL1R1 is shown in Additional file 2: Fig. S2g (*p* < 0.01). Notably, the results of behavioral tests showed that silencing IL1R1 significantly alleviated mechanical allodynia on days 14 and 15 (*p* < 0.001, Fig. 6f). Moreover, double immunofluorescence staining showed that both IL1R1 and p-p38 were expressed with IBA-1 in microglia, and the blockade of IL1R1-p-38 MAPK was consistent with the reduced fluorescence intensity of IBA-1 (Fig. 6g). In addition, the expression of IL1R1 in the TG was also inhibited (Additional file 2: Fig. S2i).

**Fig. 7:**
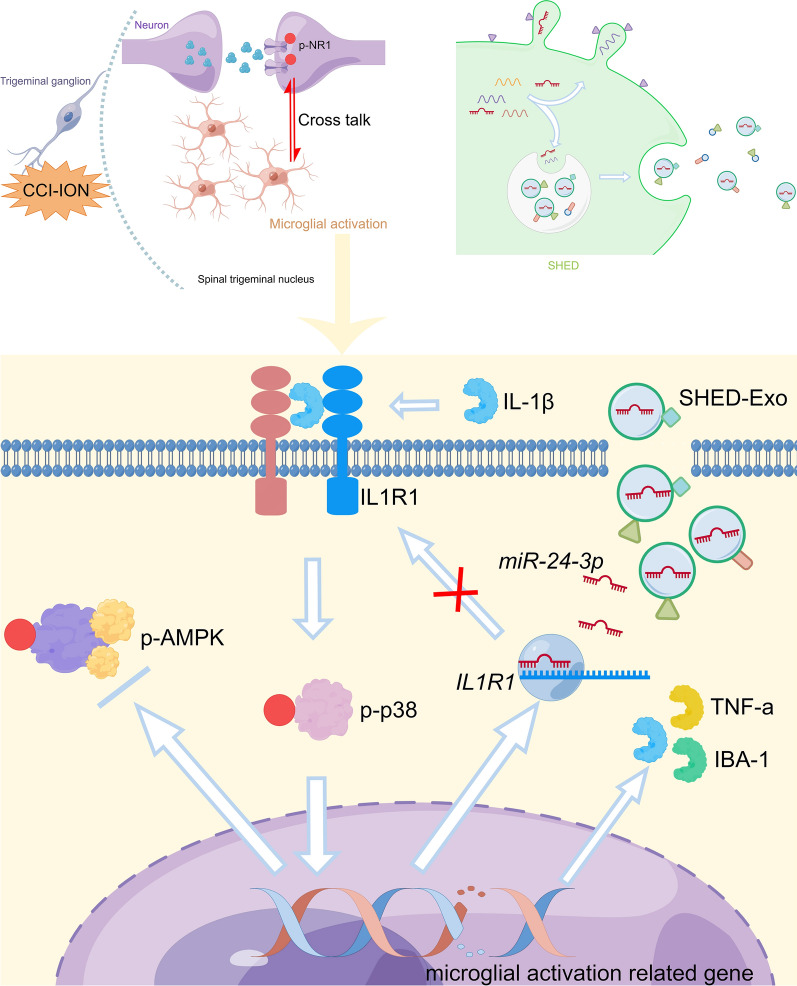
Mechanism diagram. SHED-derived exosomes encapsulated miR-24-3p and suppressed microglial activation in the STN by targeting the IL-1R1/p38 MAPK pathway, thereby reducing neuronal hypersensitivity and attenuating TN after CCI of the infraorbital nerve in mice. (This figure was drawn by Figdraw software. https://www.figdraw.com/static/index.html#/)

In general, our findings suggested that SHED-Exos transported miR-24-3p to microglia in the STN of CCI-ION mice, which suppressed microglial activation and enhanced phosphorylated AMPK, thereby inhibiting neuronal excitability and attenuating TN (Fig. 7).

## Discussion

Recently, EV-based stem cell therapy has drawn increasing attention in regulating microglial function. SHED-derived EVs present superior potential due to their accessibility [44]. Jonavice et al. reported that SHED-EVs have the ability to decrease M1 microglia while increasing M2 microglia by suppressing the NF-κB signalling pathway [21]. Similarly, Li et al. found that SHED-Exos can alleviate neuroinflammation in models of traumatic brain injury by modulating the polarization of microglia towards M1/M2 [45]. Furthermore, they also discovered that SHED-EVs promote the polarization of anti-inflammatory microglia by transferring miR-330-5p and targeting the Ehmt2/CXCL14 axis [46]. The impact of SHED-Exos on TN in relation to microglial function has not been investigated. This current study revealed, for the first time, that the administration of SHED-Exos mitigated CCI-induced TN in mice (as determined by von Frey tests and p-NR1 levels in the STN) through exosome-mediated reduction of microglial inflammation. Notably, the activation of AMPK, a crucial regulator closely associated with energy metabolism, was observed in CCI mice treated with SHED-Exos. Mechanistically, we elucidated that miR-24-3p transferred by SHED-Exos targets IL1R1-p38 MAPK signaling to exert a significant effect. 

Currently, there is a consensus in the academic community that neuropathic pain (NP) subsequent to peripheral nerve injury is intricately associated with central sensitization [47], which is characterized by an augmented nociceptive response. Infraorbital nerve ligation is a validated model for producing allodynia in the ipsilateral vibrissae area in rats [5, 27] or mice [4]. Consistently, mechanical withdrawal of CCI mice in this study was significantly reduced in a time-dependent manner and a peak on day 14 after CCI was observed. The NMDA receptor, an ionotropic receptor expressed in neurons responsible for modulating neuronal excitability, can undergo phosphorylation of its NR1 subunit by inflammatory factors (e.g., IL-1β) during the occurrence of mechanical allodynia, thereby facilitating the transmission of pain signals [48]. The in vivo findings of this study align with previous research [4, 5] indicating an increase in p-NR1 levels in the STN of CCI mice. Additionally, our study demonstrates that the administration of SHED-Exos effectively suppresses p-NR1. It is important to note that microglial activation in the STN plays a crucial role in the early stages of trigeminal central sensitization. This activation is a key driver in regulating neuronal hyperexcitability, not only through indirect mechanisms such as the release of proinflammatory factors and neuroligands but also through direct modulation of synaptic ion homeostasis [49, 50]. Moreover, microglia are also targets of these cytokines and participate in auto/paracrine interactions with astrocytes, neurons, the endothelium, and leukocyte infiltrates, thus resulting in chronic pain induction and development. Collectively, microglial activation acts as the core of ‘inflammatory storms’, which are crucial for the maintenance of TN; thus, intervention in microglial activation is a key strategy. Our results demonstrated that SHED-Exos significantly alleviated the CCI-induced mechanical pain threshold in mice and repressed microglial activation of the STN in a dose-dependent manner, as shown by the reduced expression levels of IL-1β, TNF-a and IBA-1. AMPK is well recognized as an ‘energy sensor’ and ensures cellular homeostasis due to its notable role in inhibiting biosynthesis. High energy levels (including high glucose/glycogen, inflammation, etc.) are associated with reduced AMPK activation [51, 52]. In recent decades, AMPK activated by various canonical/noncanonical regulation pathways has been regarded as a therapeutic target for NP [53]. Our results showed enhanced dephosphorylation of the α-subunit (Thr183/172) of AMPK in CCI mice, but remarkably, administration of SHED-Exos notably restored microglia to a relatively balanced energy status, thereby improving the phosphorylation level of AMPK.

Exosomes exhibit distinct biogenesis pathways and possess specific physical attributes. The phenotypic traits of SHED-Exos, including production, particle size, and distribution, may exhibit variability due to factors such as extraction techniques, storage conditions, cellular number, cellular passage, or culture conditions [34, 38]. Furthermore, the composition of exosomal cargos is contingent upon the originating cell type. To date, there is growing evidence regarding the functional role of exosome-derived miRNAs, such as their potential as biomarkers and their involvement in disease monitoring [33]. In the case of exosomes derived from mesenchymal stem cells (MSCs), miRNA cargos serve as regulators by horizontally transferring to target cells, thereby inducing phenotypic modifications and reprogramming. It is worth noting that the encapsulation of miRNAs within exosomes provides protection against degradation, enabling their effective utilization [54]. Previous studies have shown that SHED-Exo-derived miR-100-5p and miR-1246 act as tumor suppressors [55], miR-100-5p also functions as an anti-inflammatory agent [39], and miR-330-5p plays a role in regulating microglial polarization [46]. Herein, our study demonstrated the anti-inflammatory and analgesic effects of SHED-Exo-derived miR-24-3p. miR-24-3p has been reported to be expressed in other MSC-derived EVs, such as UMSC-Exos. It has been observed that Exo-derived miR-24-3p promotes M2 macrophage polarization [56, 57]. Interestingly, previous studies have established that miR-24-3p plays a role in promoting inflammation recovery [58–61]. However, the specific mechanisms by which miR-24-3p affects microglia and its potential contribution to pain relief remain largely unknown. In our current study, we conducted experiments to validate the effects of miR-24-3p on attenuating TN and inhibiting microglial activity in CCI mice. This was achieved by manipulating the expression levels of miR-24-3p in SHED-Exos, either through overexpression or downregulation. Intriguingly, the upregulation of miR-24-3p not only suppressed neuroinflammation but also remarkably heightened AMPK activation.

The bioinformatic analysis conducted in this study revealed that miR-24-3p targets *IL1R1*, a gene associated with inflammation in MAPK signaling. IL-1R1 is expressed on the cell membrane of nearly all eukaryotic cells, with varying levels of expression depending on cell type (higher expression in immune cells) [62]. The expression of IL-1R1 protein is known to regulate inflammation by facilitating the uptake of IL-1β [63]. IL1R1 has been implicated in various diseases, including liver injury [64], kidney injury [65], ischemic injury [36] and neurodegeneration [66]. Here, it was first verified that IL1R1 mediates microglial activation in TN. The p38 MAPK subfamily has been extensively studied and implicated in numerous chronic inflammatory diseases, including NP. Prior research has established that phosphorylated p38 MAPK plays a role in cell proliferation, differentiation, and inflammation, with inflammatory factors being a consequence of p38 MAPK signaling pathways [67–70]. Notably, p38 MAPK signaling is primarily linked to the microglial inflammatory response during the early stages of NP [71]. Consequently, we conducted additional investigations to assess the degree of p38 activation. Our in vitro results showed that the expression of IL1R1 and p-p38 was significantly increased in LPS-induced BV-2 cells, and their expression patterns in inflammation were consistent with those of IBA-1, IL-1β and TNF-a. However, IL1R1 had expression patterns complementary to those of miR-24-3p, suggesting that miR-24-3p was endogenously downregulated in inflammatory BV-2 cells and that silencing IL1R1 reversed miR-24-3p expression. Moreover, activated AMPK was significantly increased in IL1R1-silenced BV-2 cells. Our in vivo experiments further indicated that IL-1R1 gene silencing ameliorated TN and suppressed the expression of IBA-1 in CCI mice by blocking increased p-p38 MAPK signaling. Furthermore, our findings revealed that miR-24-3p altered the cell cycle of BV-2 cells, specifically prolonging the G0/G1 phase and shortening the S + G2/M phase. Consequently, IL1R1-silenced cells exhibited a diminished proliferation capability.

Notably, our results suggested that silencing IL1R1-p38 MAPK signaling contributed to an increase in p-AMPK, which is not equivalent to AMPK being directly regulated by p38 MAPK. It has been reported that inflammation reduces AMPK activity by ubiquitylation of LKB1, an upstream kinase of AMPK [72]. Therefore, in our study, the phosphorylation levels of AMPK in microglia were contingent upon the energy state, unless there was an intervention specifically targeting AMPK. The dephosphorylation of AMPK was observed alongside microglial activation, whereas the restoration of microglial activity led to the phosphorylation of AMPK. These findings suggest that AMPK activation may be a consequence of neuroinflammation alleviation. Conclusive evidence has demonstrated that active AMPK targets more than 100 downstream substrates [53]. For instance, the inhibition of protein synthesis occurs through the inactivation of mTORC1 [73], while the phosphorylation of PGC-1α enhances mitochondrial biogenesis [74, 75]. Although the activated MAPK pathway has been extensively studied in response to various stimuli, its interaction with AMPK remains uncertain and subject to controversy due to the intricate nature of molecular mechanisms. For example, p-AMPK may induce p38 MAPK phosphorylation in glucose uptake or cancer cell metabolism [76, 77]; nevertheless, p-AMPK prominently inhibits p-p38 levels in inflammatory microglia of TN [5]. Conversely, AMPK can also function as the downstream effector of MAPKs [78]. Thus, the interplay between p38 MAPK and AMPK requires further research.

Our initial investigation aimed to clarify the involvement of SHED-Exos in reducing TN and the underlying regulatory mechanism in microglia. We specifically highlighted the impact of exosomes containing miR-24-3p and the IL1R1/p-38 MAPK pathway. However, it is important to note that astrocyte activation plays a significant role in mediating TN maintenance. Therefore, future research will focus on examining the effect of SHED-Exos on astrocytes during the pain maintenance phase of CCI-induced TN.

## Conclusions

Collectively, our findings demonstrate that SHED-Exos effectively mitigate microglial activation and neuronal hyperactivity in the STN of mice with CCI-ION, potentially through the involvement of the miR-24-3p/IL1R1/p38 MAPK axis. Additional mechanisms may also contribute to the alleviation of TN, suggesting that SHED-Exos hold promise as a modulator of allodynia and neuroinflammation, as well as a therapeutic approach for TN and other neuropathic disorders. Furthermore, it is imperative to elucidate the underlying mechanisms by which SHED-Exos preserve or enhance mitochondrial function in attenuating TN.

Abbreviations used throughout the article were shown in Additional file 6: Table S4.

### Supplementary Information

**Additional file 1: Fig. S1** Characteristics of SHEDs. a (i) Representative panoramic radiograph indicating a retained deciduous tooth in a mixed dentition, (ii) (iii) primary and passage 3 SHEDs (scale bar: 100 μm). b SHED surface molecules were analysed by flow cytometry. c (i)-(iv) ALP/ARS/Alcian blue/Oil red O staining of induced SHEDs and control SHEDs (scale bar: 100 μm), (v) immunofluorescence detection of neuronal markers in induced SHEDs and control SHEDs (scale bar: 25 μm).

**Additional file 2: Fig. S2** a PKH26-labelled SHED-Exos were distributed in the ipsilateral TG (scale bar: 100 μm). b Cell cycle of inflammatory BV-2 cells transfected with NC/mimic of miR-24-3p. c Cell cycle of inflammatory BV-2 cells transfected with INC/Inhibitor of miR-24-3p. d Levels of IL1R1 mRNA in LPS-stimulated BV-2 cells with or without SHED-Exos. e Expression pattern of miR-24-3p in BV-2 cells after LPS stimulation. f Levels of miR-24-3p in LPS-stimulated BV-2 cells with or without si-IL1R1. g Expression of IL1R1 in CCI mice with or without si-IL1R1 (#2). h-Cell cycle of inflammatory BV-2 cells transfected with si-NC/si-IL1R1 (#2). i Immunofluorescence indicated that administration of si-IL1R1 led to a decrease in IL1R1 in the TG (scale bar: 100 μm). (*p < 0.05, **p < 0.01, ***p < 0.001 vs. Control/Sham groups; #p < 0.05, ##p < 0.01, ###p < 0.001 vs. LPS/CCI groups)

**Additional file 3: Table S1.** Comparison of cell proliferation between NC and Mimics groups

**Additional file 4: Table S2.** Comparison of cell proliferation between INC and Inhibitor groups

**Additional file 5: Table S3.** Comparison of cell proliferation between si-NC and si-IL1R1 groups

**Additional file 6: Table S4.** Abbreviations used throughout the article

## Data Availability

Data used and analyzed during the current study are available from the corresponding author on reasonable request.
