# Supplementary figures and images for: SHED-derived exosomes attenuate trigeminal neuralgia after CCI of the infraorbital nerve in mice via the miR-24-3p/IL-1R1/p-p38 MAPK pathway
Source: J Nanobiotechnology. 2023 Nov 29;21:458. doi: 10.1186/s12951-023-02221-6 (PMC10685568; doi:10.1186/s12951-023-02221-6)

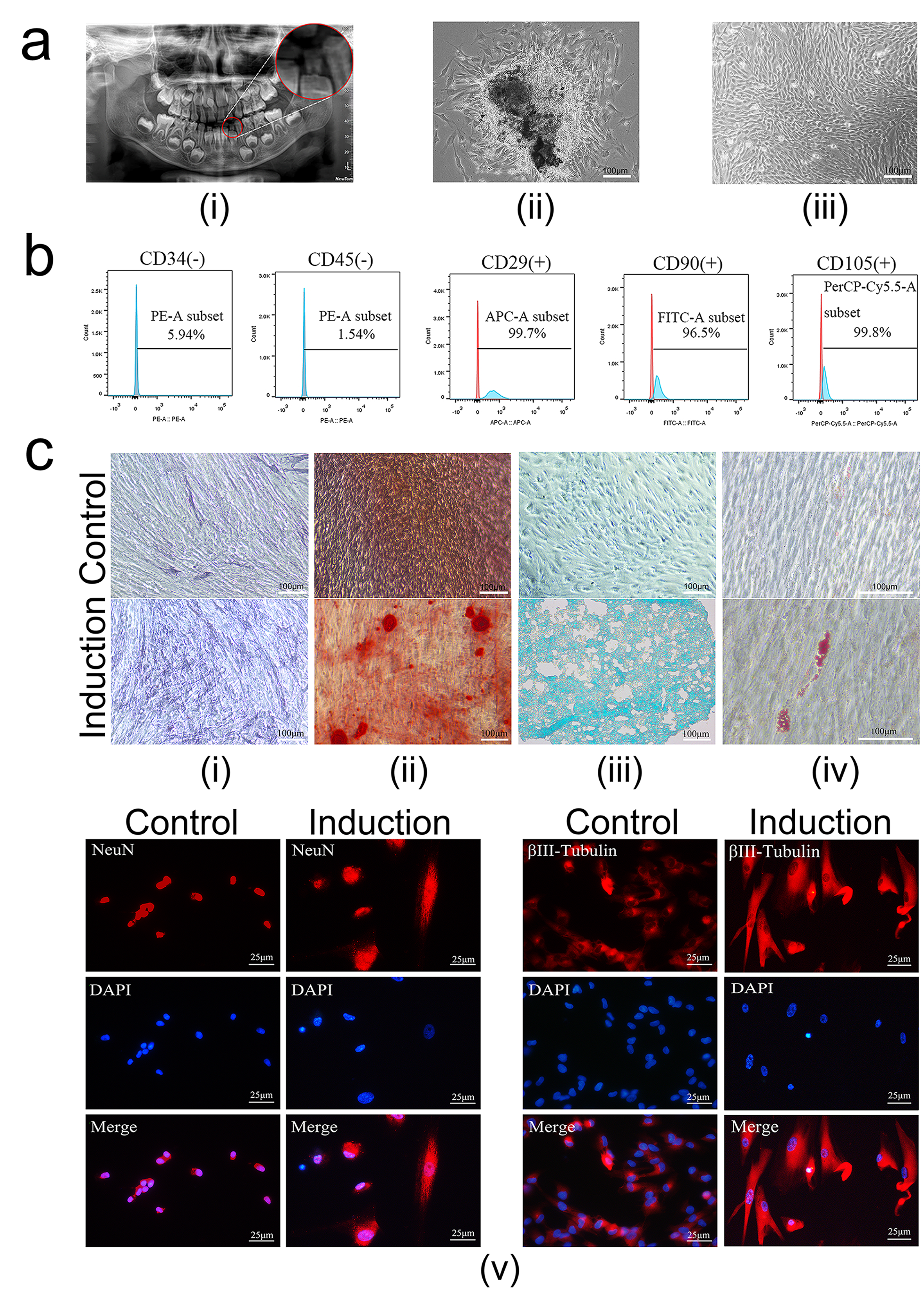

Supplement: Supplementary file 1 — Additional file 1: Fig. S1 Characteristics of SHEDs. a (i) Representative panoramic radiograph indicating a retained deciduous tooth in a mixed dentition, (ii) (iii) primary and passage 3 SHEDs (scale bar: 100 μm). b SHED surface molecules were analysed by flow cytometry. c (i)-(iv) ALP/ARS/Alcian blue/Oil red O staining of induced SHEDs and control SHEDs (scale bar: 100 μm), (v) immunofluorescence detection of neuronal markers in induced SHEDs and control SHEDs (scale bar: 25 μm). [file 12951_2023_2221_MOESM1_ESM.tif]

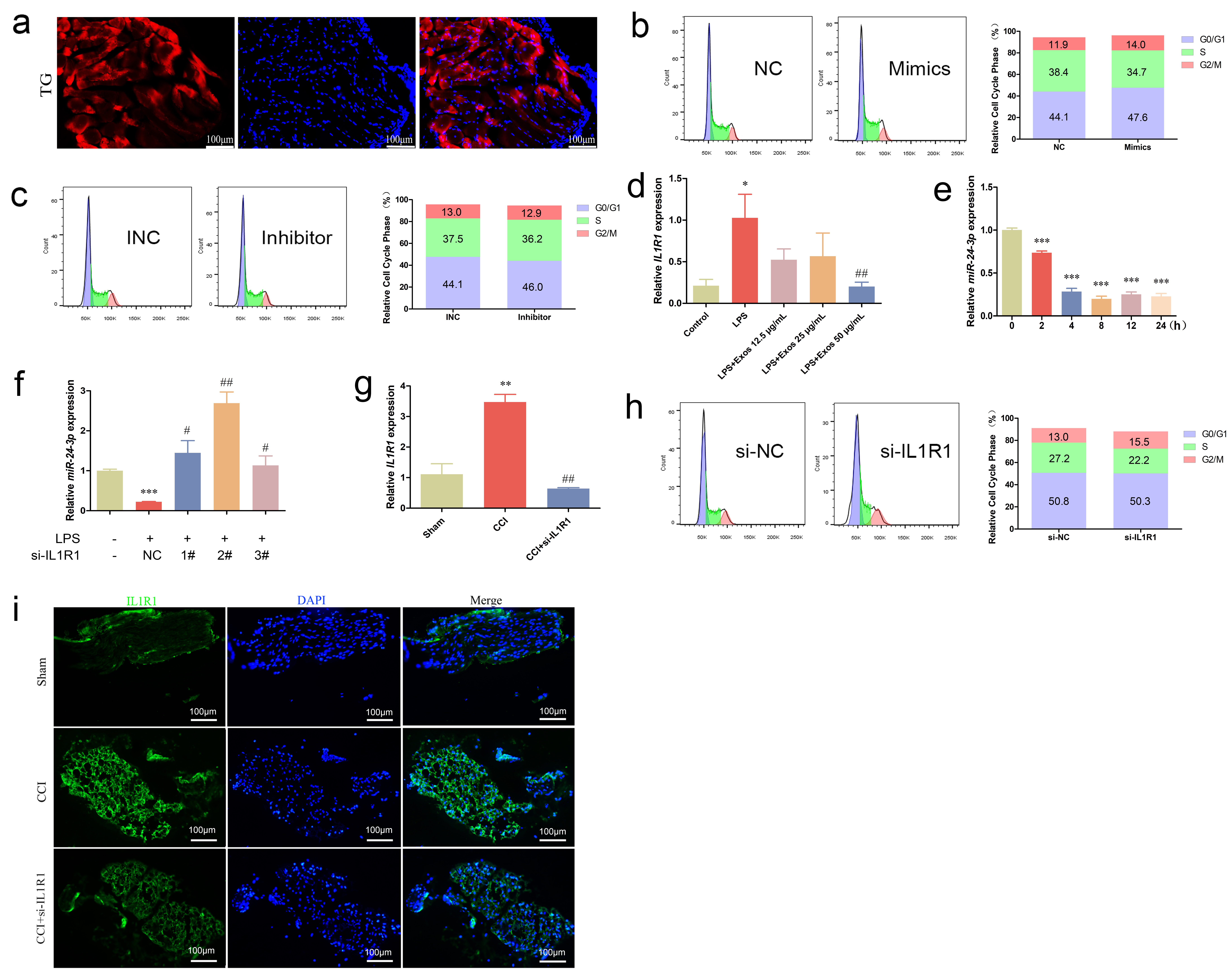

Supplement: Supplementary file 2 — Additional file 2: Fig. S2 a PKH26-labelled SHED-Exos were distributed in the ipsilateral TG (scale bar: 100 μm). b Cell cycle of inflammatory BV-2 cells transfected with NC/mimic of miR-24-3p. c Cell cycle of inflammatory BV-2 cells transfected with INC/Inhibitor of miR-24-3p. d Levels of IL1R1 mRNA in LPS-stimulated BV-2 cells with or without SHED-Exos. e Expression pattern of miR-24-3p in BV-2 cells after LPS stimulation. f Levels of miR-24-3p in LPS-stimulated BV-2 cells with or without si-IL1R1. g Expression of IL1R1 in CCI mice with or without si-IL1R1 (#2). h-Cell cycle of inflammatory BV-2 cells transfected with si-NC/si-IL1R1 (#2). i Immunofluorescence indicated that administration of si-IL1R1 led to a decrease in IL1R1 in the TG (scale bar: 100 μm). (*p < 0.05, **p < 0.01, ***p < 0.001 vs. Control/Sham groups; #p < 0.05, ##p < 0.01, ###p < 0.001 vs. LPS/CCI groups) [file 12951_2023_2221_MOESM2_ESM.jpg]
