# Supplementary material for: SHED-derived exosomes attenuate trigeminal neuralgia after CCI of the infraorbital nerve in mice via the miR-24-3p/IL-1R1/p-p38 MAPK pathway
Source: J Nanobiotechnology. 2023 Nov 29;21:458. doi: 10.1186/s12951-023-02221-6 (PMC10685568; doi:10.1186/s12951-023-02221-6)
Supplement: Supplementary file 3 — Additional file 3: Table S1. Comparison of cell proliferation between NC and Mimics groups [file 12951_2023_2221_MOESM3_ESM.docx]

| **Table S1.** Comparison of cell proliferation between NC and Mimics groups | | | |
| --- | --- | --- | --- |
| **Data analyzed** |  |  |  |
|  | G0/G1 | S+G2/M | Total |
| NC | 7661 | 8739 | 16400 |
| Mimics | 8577 | 8775 | 17352 |
| Total | 16238 | 17514 | 33752 |
| **Statistical results of Chi-square test** |  |  |  |
| Chi-square, df | 24.91, 1 | | |
| P value | < 0.0001 | | |
