# Supplementary material for: SHED-derived exosomes attenuate trigeminal neuralgia after CCI of the infraorbital nerve in mice via the miR-24-3p/IL-1R1/p-p38 MAPK pathway
Source: J Nanobiotechnology. 2023 Nov 29;21:458. doi: 10.1186/s12951-023-02221-6 (PMC10685568; doi:10.1186/s12951-023-02221-6)
Supplement: Supplementary file 4 — Additional file 4: Table S2. Comparison of cell proliferation between INC and Inhibitor groups [file 12951_2023_2221_MOESM4_ESM.docx]

| **Table S2.** Comparison of cell proliferation between INC and Inhibitor groups | | | |
| --- | --- | --- | --- |
| **Data analyzed** |  |  |  |
|  | G0/G1 | S+G2/M | Total |
| INC | 8481 | 8553 | 17034 |
| Inhibitor | 7861 | 9002 | 16863 |
| Total | 16342 | 17555 | 33897 |
| **Statistical results of Chi-square test** |  |  |  |
| Chi-square, df | 34.14,1 | | |
| P value | < 0.0001 | | |
