# Supplementary material for: SHED-derived exosomes attenuate trigeminal neuralgia after CCI of the infraorbital nerve in mice via the miR-24-3p/IL-1R1/p-p38 MAPK pathway
Source: J Nanobiotechnology. 2023 Nov 29;21:458. doi: 10.1186/s12951-023-02221-6 (PMC10685568; doi:10.1186/s12951-023-02221-6)
Supplement: Supplementary file 5 — Additional file 5: Table S3. Comparison of cell proliferation between si-NC and si-IL1R1 groups [file 12951_2023_2221_MOESM5_ESM.docx]

| **Table S3.** Comparison of cell proliferation between si-NC and si-IL1R1 groups | | | |
| --- | --- | --- | --- |
| **Data analyzed** |  |  |  |
|  | G0/G1 | S+G2/M | Total |
| si-NC | 7190 | 5690 | 12880 |
| si-IL1R1 | 5656 | 4239 | 9895 |
| Total | 12846 | 9929 | 22775 |
| **Statistical results of Chi-square test** |  |  |  |
| Chi-square, df | 4.069,1 | | |
| P value | 0.0437 | | |
