# Supplementary material for: SHED-derived exosomes attenuate trigeminal neuralgia after CCI of the infraorbital nerve in mice via the miR-24-3p/IL-1R1/p-p38 MAPK pathway
Source: J Nanobiotechnology. 2023 Nov 29;21:458. doi: 10.1186/s12951-023-02221-6 (PMC10685568; doi:10.1186/s12951-023-02221-6)
Supplement: Supplementary file 6 — Additional file 6: Table S4. Abbreviations used throughout the article [file 12951_2023_2221_MOESM6_ESM.docx]

| **Table S4. Abbreviations used throughout the article** | |
| --- | --- |
| **Term** | **Abbreviation** |
| Spinal trigeminal nucleus | STN |
| Trigeminal neuralgia | TN |
| Adenosine monophosphate-activated protein kinase | AMPK |
| N-methyl-D-aspartate receptor1 | NMDAR1, NR1 |
| Stem cells from human exfoliated deciduous teeth | SHED |
| SHED derived exosomes | SHED-Exos |
| Chronic constriction injury | CCI |
| Infraorbital nerve | ION |
| Lipopolysaccharide | LPS |
| Interleukin-1 receptor type 1 | IL1R1 |
| Quantitative real-time polymerase chain reaction analysis | qRT-PCR |
| Neuropathic pain | NP |
| Trigeminal ganglion | TG |
| Protein kinase C | PKC |
| Protein kinase A | PKA |
| Selective Nerve root Injection | SNI |
| Chloro fluoro aniline | CFA |
| Interleukin-1β | IL-1β |
| Tumor necrosis factor-α | TNF-α |
| Matrix metalloproteinase | MMP |
| Colony-stimulating factor 1receptor | CSF1R |
| Conditioned media | CM |
| Extracellular vesicles | EV |
| Transmission electron microscope | TEM |
| Interleukin-1 receptor antagonist | (IL-1Ra) |
| Phosphate buffered saline | PBS |
| Alpha minimum essential medium | α-MEM |
| Fetal bovine serum | FBS |
| Alkaline phosphatase | ALP |
| Alizarin red staining | ARS |
| Dulbecco’s modified Eagle’s high-glucose medium | DMEM |
| Nanoparticle tracking analysis | NTA |
| Paraformaldehyde | PFA |
| Cycle threshold | CT |
| Immunocytochemistry | ICC |
| Cell counting kit-8 | CCK-8 |
| Propidium iodide | PI |
| One-way analysis of variance | One-way ANOVA |
| Two-way analysis of variance | Two-way ANOVA |
